# Quality Characteristics Changes of the Fat Portion of Chinese Bacon During Processing Based on Physicochemical Properties and Microstructure Studies

**DOI:** 10.3390/foods13233821

**Published:** 2024-11-27

**Authors:** Chuxin Liu, Enqi He, Peitao Fu, Leli He, Lei Zhou, Aihua Lou, Yan Liu, Haohua Fu, Qingwu Shen, Jie Luo, Wei Quan

**Affiliations:** 1 College of Food Science and Technology, Hunan Agricultural University, Changsha 410128, Chinaluojie@hunau.edu.cn (J.L.); 2 Tangrenshen Group Co., Ltd., Zhuzhou 412000, China

**Keywords:** Chinese bacon, low-temperature smoking treatment, fat portion, melting point, crystallization behavior

## Abstract

In order to elucidate the development of quality properties in the fat portion of Chinese bacon during low-temperature smoking (LTS), raw pork was cured for five days, followed by infusion with smoked liquid and a subsequent ten-day smoking period characterized by alternating high and low-temperature conditions. The physicochemical characteristics and microstructures of the fat portion of the Chinese bacon were examined at three stages: the raw meat stage (Control), the curing stage (C3d and C5d), and the smoking stage (S5d and S10d). The results showed that LTS increased the hardness, transparency, and b* value of bacon fat. The increased contents of neutral lipids and free fatty acids, increased activities of neutral lipase and lipoxygenase, and increased peroxide and thiobarbituric acid reactive substance value indicated significant lipolysis and lipid oxidation of bacon fat during LTS. After the treatment, a decreased melting point and increased β′- and β-type fat crystal formation were observed in the fat portion. Moreover, the treatment led to disruption of the adipocyte membrane structure. Therefore, the destruction of adipocytes after lipolysis and lipid oxidation during low-temperature smoking treatment might contribute to the development of quality properties of bacon fat portions. Precise control of temperature and time enhances the stability of the fat portion of bacon, thereby improving quality characteristics such as texture and appearance.

## 1. Introduction

Traditional Chinese bacon is a Chinese delicacy that has long been highly regarded by the Chinese population for its simple preparation, storage stability, rich flavor, and appealing color. The pig is slaughtered and cut into pieces, after which salt and other spices are applied to the surface of the pork pieces, and the meat is cured for approximately 1 week. Subsequently, the cured pork is exposed to wood-burning smoke for 20 days [1,2,3,4]. The final product, known as traditional Chinese bacon, is characterized by red or yellow skin, white fat, and brownish-red muscle [5]. After cooking and slicing, the bacon appears brightly colored, and its fat portion appears transparent and shiny [2,4]. The product possesses a robust smoky flavor profile and is relatively free of greasiness. In traditional Chinese bacon production, fat plays an essential role. The presence of fat in bacon prevents excessive water and nutrient loss during the curing and smoking stages and contributes to the juiciness and tenderness of the product. Fat plays an essential role in the development of the characteristic flavor of bacon [6].

Hydrolysis and oxidation of fat occur during the processing of traditional Chinese bacon. Esterases hydrolyze fats and phospholipids to produce free fatty acids. Oxidation of free fatty acids results in the formation of numerous hydroperoxides, which undergo a multitude of decomposition pathways, ultimately producing a plethora of volatile compounds or aromatic precursors [7]. A previous study [8] discovered that phospholipid hydrolysis can facilitate the oxidation of the fat portion of bacon. There exist three pathways for fat oxidation: auto-oxidation, which is mediated by hydroxyl radicals; enzymatic oxidation, which mainly occurs through endogenous lipoxygenase (LOX); and photosensitized oxidation, which is induced by mono-linear oxygen [9]. Auto-oxidation was found to be the main cause of fat oxidation in bacon [10], followed by LOX-mediated oxidation of polyunsaturated fatty acids in bacon to generate hydroperoxides [11]. Furthermore, photosensitive oxidation of fat may be more prevalent in sun-dried bacon. Hydrolysis and oxidation of fat during the processing of bacon are influenced by numerous factors, such as the processing method, temperature, humidity, and microbiota [12]. It is evident that further research is warranted to fully comprehend the alterations in fat contents that occur during the processing of foods. Based on the previous research, it is evident that traditional Chinese bacon must contain a substantial amount of fatty tissue because the product would not be considered traditional without fatty tissue.

Consumption of traditional Chinese preserved meat may be associated with certain health risks. Nitrites and benzo(a)pyrene produced during the curing and smoking of bacon are considered carcinogens, which increase the risk of cancer. Consequently, to reduce the levels of certain substances, such as salt, nitrite, and benzo(a)pyrene in bacon and enhance the efficiency of industrialized bacon production, our previous study examined low-temperature smoking treatment as an alternative to the traditional processing method [13]. This previous study revealed that the bacon produced via low-temperature smoking treatment not only had lower amounts of harmful substances, such as benzo(a)pyrene but also showed improved organoleptic quality. In particular, the fat portion of the cooked bacon product appeared transparent and almost completely melted at oral temperatures. Nevertheless, the precise mechanism by which the fat portion of the bacon produced via low-temperature smoking treatment becomes transparent after heating remains unclear.

This study aimed to examine the alterations in the quality, composition, and characteristics of the fat portion of bacon during low-temperature smoking treatment and elucidate the mechanisms underlying the transparent appearance of the fat portion after heating.

## 2. Materials and Methods

### 2.1. Preparation of Bacon Samples

Pork meat was extracted from the loin and belly of a crossbred pig (Duroc × Landrace × Yorkshire). First, thoroughly wash and dry the fresh pork belly. Subsequently, cut the pork into uniform strips measuring approximately 40 cm in length, 6 cm in width, and 6 cm in height (approximately 1.44 kg). Finally, divide the strips into five groups, ensuring that each group contains three strips. The pork belly pieces were processed according to a novel procedure known as Hunan bacon production, which included curing and smoking stages as described in our previous study. During the curing stage, the pork belly pieces were cured with 3.5% dry salt, 0.01% sodium nitrite, and 0.15% sodium D-isoascorbate at 4 °C for 5 days. During the smoking process, pork pieces are placed in a constant temperature and humidity box (BSC-250, Shanghai Baoxun Medical Biotechnology Co., Shanghai, China). Liquid smoke (Smokez C-10, Red Arrow, Cambridge, MA, USA), measured at 2 g, is uniformly applied to the meat using a spraying technique, with three applications conducted daily. In the liquid smoking phase, alternating high and low-temperature cycles are implemented every 8 h. Specifically, the temperature is maintained at 55 °C with a humidity level of 70% for a duration of 4 h, followed by a temperature of 4 °C with no restrictions on humidity. This new procedure shortened the processing time of bacon to 15 days. The fat portions of raw meat and bacon processed at 3, 5, 10, and 15 days were collected and stored at −80 °C until further analysis. The experimental samples were named C3d and C5d after curing for 3 and 5 days, respectively, and S5d and S10d after smoking for 5 and 10 days, respectively.

### 2.2. Color and Texture Profile Analysis of the Fat Portion of Bacon

The colorimeter (CS-580, Hangzhou Chroma Technology Co., Hangzhou, China) was calibrated using black and white standards, with calibration parameters for black being lightness (L*), redness (a*), and yellowness (b*) values of 0, and for white being L*, a*, and b* values of 87.62, −0.77, and 1.21, respectively. Measurements were conducted under a D65 light source at a 10° angle. The fat portion of bacon was securely clamped at the measuring mirror opening, and measurements were taken at three different points on three separate occasions. The fat portion of bacon was sectioned into dimensions of 2 × 2 × 1 cm. The texture properties, including hardness, springiness, cohesiveness, and resilience, were assessed using the texture analyzer (TA. XT Plus, Surface Measurement Systems, Wembley, UK). The following parameters were established: compression ratio of 50%, pre-test speed of 2.00 mm/s, test speed of 1.00 mm/s, return speed of 1.00 mm/s, trigger point load of 5 g, and probe type P/36R.

### 2.3. Extraction and Separation of Neutral Lipids, Free Fatty Acids, and Phosphatide

Lipids were extracted using the Storrustlokken extraction method [14]. Briefly, 2.0 g of fat portions were homogenized with 25 mL of CHCl_3_:MeOH (2:1, *v*/*v*) for 1 min using a homogenizer (T10, IKA Works GmbH & Co., Staufen im Breisgau, Germany). Then, cold methyl tert-butyl ether (1:5, *w*/*v*) was added, and the mixture was shaken, followed by centrifugation for 10 min at 5000 rpm and 4 °C. The organic layer was isolated by filtering the supernatant through a 100 mg solid-phase extraction column. Neutral lipids, free fatty acids, and phosphatides were separated using 5 mL of a chloroform-isopropanol solution (2:1, *v*/*v*), 5 mL of an acetic acid-ether solution (2%, m/m), and a methanol-HCl solution (9:1, *v*/*v*), respectively. The resulting extracts were collected in individual centrifuge tubes, evaporated with nitrogen gas, and weighed [15]. It is essential to calculate the percentage composition of each lipid component in relation to the total fat, which is defined as the sum of the three components.

### 2.4. Fatty Acid Composition Analysis

According to Benet et al. [16], 0.1 g of total lipids underwent a conversion process procedure to produce fatty acid methyl esters (FAMEs). Following this, the sample was allowed to cool to room temperature before 3 mL of hexane and 10 mL of saturated NaCl solution were added, and the mixture was shaken for 10 s before being left to sit for 1 h. The upper phase underwent filtration using a 0.22-μm membrane and was then transferred to a sample vial for analysis utilizing an Agilent GC 7890A gas chromatograph equipped with a flame ionization detector and an DB-23 column (Agilent Technologies, Inc., Santa Clara, CA, USA). A total of 1 μL of the sample was injected with a split ratio of 10:1, while the injector and detector temperatures were set at 250 °C. The oven temperature was initially set at 170 °C for 3 min, then increased at a rate of 20 °C/min to 200 °C, and further increased at a rate of 3.5 °C/min to 210 °C. It was then raised to 230 °C at a rate of 1.5 °C/min and maintained for 13 min. By comparing the retention times of the samples with those of the standard (Supelco, Bellefonte, PA, USA), the identification of fatty acids is achieved. The results are presented as a percentage of the total fatty acid methyl esters detected.

### 2.5. Crystallization and Melting of the Fat Portion of Bacon

#### 2.5.1. Differential Scanning Calorimetry (DSC)

DSC was used to conduct isothermal crystallization and melting experiments on the fat portion. (Mettler Toledo, Greifensee, Switzerland). About 0.8 mg of fat sample was moved into an aluminum pan and tightly sealed. To construct the melting curve, the sample was maintained at −40 °C for 10 min and heated to 80 °C at a rate of 5 °C/min. The sample was maintained at 80 °C for 10 min to eliminate the crystal structure, and crystallization curves were constructed after cooling the samples to −40 °C at a rate of 5 °C/min [17].

#### 2.5.2. X-Ray Diffraction (XRD)

Overall, 30 g of subcutaneous fat sample was churned and placed at 50 °C. The fat sample was ground and filtered after 3 h. The sample was then placed at 50 °C for 30 min to eliminate historical crystallization. Finally, the samples were stored at 4 °C for 12 h for subsequent analysis. To obtain the diffraction patterns, X-ray analysis was performed using an XRD instrument (6000 X, Shimadzu, Japan) operating at 40 kV and 30 mA (Cu-K alpha, l = 1.5418 Å). Angle X-ray scans (15° < 2θ < 30° at 2°/min) were performed at 20 °C [18].

### 2.6. Lipid Oxidation Analysis

TBARS and POV were determined according to the procedure described by the manufacturer of commercial kits (Solarbio, Beijing, China). The TBARS value was expressed as milligrams of malondialdehyde equivalents per kilogram of sample, whereas the POV was expressed as milli-equivalents of peroxide per kilogram of lipid sample.

### 2.7. Determination of Lipase and LOX Activities

The activities of lipases, including neutral lipase, phospholipase, and acid lipase, were measured according to a previously described procedure [19]. The LOX activity in the fat portion of bacon was determined according to a previously reported method [11], and the results were expressed as U g^−1^ fat.

### 2.8. Morphology of the Adipocyte Membrane in the Fat Portion

A cryosectioner was used to slice the subcutaneous fat portion of the bacon sample into 10-μm sections. The sections were dried and stained with 1 mg/mL sulforhodamine B for 5 min, after which they were washed with chloroform to remove the excess staining solution. Finally, the sections were observed under a polarized light microscope (200× magnification; DP27, Olympus Corporation, Tokyo, Japan).

### 2.9. Statistical Analysis

All experiments were conducted in triplicate, and the final results are presented as mean ± standard deviation. To assess the differences among various treatments, we employed the Generalized Linear Mixed Model (GLMM) using SAS 9.0 software (SAS Institute Inc., Cary, NC, USA) for variance analysis. In this model, the fat portion of bacon at different time points during the processing was treated as a fixed effect, while individual variations and experimental batches were considered as random effects. The Tukey–Kramer method, with the adjusted PDIFF option, was utilized for multiple comparisons to derive the least squares mean, thereby ensuring the accuracy and reliability of the results.

## 3. Results

### 3.1. Changes in the Color and Texture Profile of the Fat Portion of Bacon During LTS

After LTS, the changes in the fat portion of bacon greatly contribute to the bacon quality. As shown in Table 1, the fat portion was characterized by a light amber color, dense tissue, and good elasticity. Specifically, the L* of the fat portion showed a decreasing trend during treatment, whereas the b* and a* values of the fat portion significantly increased after treatment. Curing and smoking showed different effects on the color of the fat portion of bacon, with smoking treatment exerting a greater impact on L* and b* values, decreasing from 78.82 to 52.45 and increasing from 6.02 to 15.08, respectively. These changes were mainly related to the color of the liquid smoke.

The sensory quality of bacon is greatly affected by the texture of the fat tissue, as the fat tissue acts like an elastic solid that can endure mechanical stress. Table 1 also shows the changes in the textural properties of the fat portion during treatment. Hardness and cohesiveness were found to be the most important indicators of the fat portion of bacon quality, the values of which significantly increased after treatment from 4558 to 17,836 and 0.63 to 0.87, respectively. Compared with raw meat fat, the springiness and resilience of the fat portion of bacon altered significantly (*p* < 0.05). Based on the effects of curing and smoking treatments on the textural quality of the fat portion, it was evident that although curing significantly affects the hardness of the fatty meat, smoking has a more substantial impact on the overall meat quality. The highest values of hardness, cohesiveness, and resilience were observed in the experimental sample S10d. According to previous research, the changes in the textural properties of fat samples during treatment were mainly related to a series of physical changes and chemical reactions [20].

### 3.2. Changes in the Fat Composition and Fatty Acid Content in Traditional Chinese Bacon During LTS Treatment

Changes in the contents of neutral lipids, free fatty acids, and phosphatide during treatment may reveal the extent of oxidation and hydrolysis of fat and significantly affect the color, texture, and flavor quality of bacon. Elevated concentrations of free fatty acids can result in undesirable flavors and may expedite the oxidation process of food, thereby reducing its shelf life. Additionally, high levels of free fatty acids can adversely affect the texture, rendering it greasy or loose. The neutral lipid contents in the fat portion of bacon at different processing time points are shown in Table 2. During treatment, compared with raw meat, the content of neutral lipids in bacon meat gradually increased and then altered significantly (*p* < 0.05) in both groups; these results were consistent with those of a previous study on dry-cured ham, which showed that the addition of NaCl affected the content of neutral lipid [15]. Moreover, compared with the curing treatment, the neutral lipid content increased significantly during the smoking treatment, reaching 77.79 g/100 g and 82.45 g/100 g in experimental samples S5d and S10d, respectively. The phosphatide content also altered significantly (*p* < 0.05) during treatment. The phosphatide content decreased rapidly from 26.2 g/100 g to 18.63 g/100 g after curing, whereas it reached 6.59 g/100 g after smoking for 10 days; these findings were similar to those of a previous study [15].

During fat decomposition, triglycerides undergo hydrolysis, resulting in the formation of glycerol and fatty acids; the content of free fatty acids largely depends on the rates at which they are formed and decomposed, which are key processes of lipid hydrolysis and oxidation. As shown in Table 2, the free fatty acid content in raw meat was 4.04 g/100 g, which increased significantly (*p* < 0.05) to 10.96 g/100 g after treatment, indicating a greater rate of formation than that of decomposition. This finding was consistent with that of reduced phosphatide content, as phospholipid hydrolysis was also found to be an important approach to producing fatty acids. The effects of smoking and curing on the content of free fatty acids are similar, with the content of free fatty acids increasing to 2.79 g/100 g and 3.77 g/100 g, respectively.

Moreover, the fatty acid composition of fat tissue is strongly linked to the quality of the product, including factors such as melting point, hardness, oxidation stability, nutritional value, and flavor [21]. As shown in Table 3, of the 24 types of fatty acids, 11 were saturated fatty acids (SFAs), and 13 were unsaturated fatty acids (UFAs). SFAs were the main fatty acids (46.78%) in raw fats, and the proportions of mono-UFAs (MUFAs) and poly-UFAs (PUFAs) were 32.22% and 20.88%, respectively. Palmitic acid (30.98%), oleic acid (26.85%), and linoleic acid (18.36%) were the major components of SFAs, PUFAs, and MUFAs in the raw fat portion. This finding is consistent with that of a previous study that investigated the fatty acid composition of traditional Chinese bacon [8]. After curing and smoking, the fatty acid contents were found to be similar across various samples. Specifically, the SFA and MUFA contents in the fat tissue decreased after curing, whereas the PUFA content significantly increased from 20.88% to 23.57% (*p* < 0.05), with linoleic acid being the most significantly altered PUFA, whose content increased from 18.36% to 21.03%. A previous study [22] also showed that the PUFA content of Xuanwei ham significantly increased during curing owing to the salt-induced inhibition of fat oxidation. However, an opposite trend in the fatty acid profile was observed in the fat portion of smoked bacon samples, with the SFA content significantly increasing (*p* < 0.05) from 44.82% to 47.03%; moreover, the PUFA content in bacon samples was significantly lower (19.25%) than that in cured samples (23.57%) (*p* < 0.05). The results were consistent with those of a previous study, which showed a significantly decreased PUFA content in Iberian ham that was induced by fat oxidation [23]. A previous study indicated that smoking treatment is a robust promoter of lipid oxidation during bacon production [20]. As a result of their unsaturated molecular structure, UFAs are converted to SFAs through oxidation, which promotes the propagation of free radicals.

### 3.3. Changes in the Melting and Crystallization Properties of Traditional Chinese Fat Portion of Bacon During Treatment

**Figure 1:**
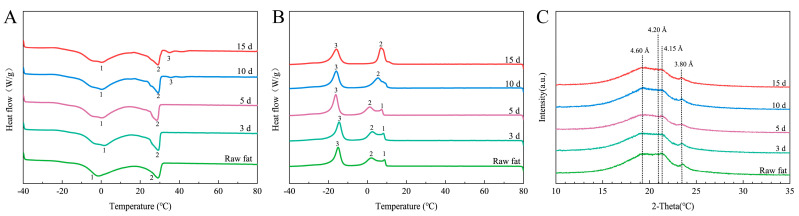
Changes in the melting and crystallization properties of fat portion of bacon during LTS. (**A**) The melting curves (measured from −40 °C to 80 °C). (**B**) Crystallization curves (measured from 80 °C down to −40 °C). (**C**) Wide-angle X-ray diffractogram of fat portion of bacon.

According to previous research, the melting point and crystallization behavior of fat tissue are closely related to its chemical structure, especially the composition of fatty acids and triacylglycerols. Therefore, this study further investigated the changes in the crystallization and melting properties of the fat portion of bacon. The DSC curves of the fat portion of bacon during heating from −40 °C to 80 °C are shown in Figure 1A. In the melting curve (Figure 1A), three endothermic peaks (1, 2, and 3) were observed, and the triacylglycerols observed at Peaks 1 and 2 were mostly melted. As shown in Table 4, the onset melting temperature of Peak 1 significantly increased from −26.63 °C to −24.01 °C, and the highest temperature of Peak 2 was between 14.58 °C and 16.45 °C, Peak 3 might indicate the melting temperature of trisaturated triacylglycerols, as it has the highest melting point. This could be due to the lower melting temperature of triunsaturated triacylglycerol. However, the Peak 3 temperatures were only observed in the control and C3d samples between 31.53 °C and 32.09 °C. The disappearance of peak 3 in the melting profiles of the fat portion during smoking treatment revealed that UFAs may be oxidized to SFAs or other secondary products, consistent with our abovementioned results.

In the crystallization curve, three exothermic peaks (1, 2, and 3) were observed (Figure 1B). As shown in Table 4, the onset crystallization temperature of peak 1 decreased significantly from 10.49 °C to 8.54 °C, and peak 1 merged with peak 2 in the liquid smoking stage. This change significantly increased the onset crystallization temperature of peak 2 from 8.54 °C in the curing stage to 13.93 °C in the liquid smoking stage. Therefore, the change in onset crystallization temperature is consistent with the change in the SFA contents. Conversely, the highest temperature of peak 3 varied between −12.52 °C and −9.55 °C, which may be due to the change in UFA contents.

Regarding fat crystallization, the state of the fat crystals in the fat portion of bacon during treatment was also investigated. The fat crystals included α, β′, and β types, with α-type crystals being unstable and having the lowest melting point [24,25]. The crystalline form of the fat at 4 °C was determined based on the short spacing of XRD patterns. As shown in Figure 1C, diffraction peaks were observed at 3.8, 4.15, and 4.6 Å for all groups; in SFAs, the characteristic short spacing was 4.15 Å for α-type crystals, 4.2 and 3.8 Å for β′-type crystals, and 4.6 Å for β-type crystals [26], which indicated that all processed fats were capable of forming α-, β′, and β-type crystals. The diffraction peak at 4.15 Å gradually decreased, indicating that the formation of α-type crystals gradually decreased during treatment. The diffraction peak at 3.8 Å decreased and then increased, indicating a decrease followed by an increase in the formation of β′-type crystals, with the minimum value observed in the experimental sample C5d. The diffraction peak at 4.60 Å gradually increased, indicating an increase in the formation of β-type crystals during treatment.

The melting point of the fat portion of bacon was also determined based on the fatty acid composition, and SFAs had a high melting point and showed the highest crystallinity. In the fat portion of sample S10d, the proportions of both SFAs and UFAs were close to 50%; therefore, two significant peaks with similar peak areas were observed in the melting curve, with the peak near 0 °C representing UFA and the other peak representing SFA. Similarly, two peaks of similar sizes were observed in the crystallization curve. Upon heating, the UFAs in the fat portion melted first, followed by SFAs. However, compared with raw fat, there was no significant change in the SFA content of the fat portion of bacon, but a significant increase in the MUFA content and a significant decrease in the PUFA content were noted in experimental sample S10d. The UFAs were already in a liquid state at room temperature and were thus excluded. Therefore, the reason for the transparent appearance of the fat portion of the bacon after heating was not due to changes in the fatty acid composition during treatment.

### 3.4. Changes in Lipase Activities in Traditional Chinese Bacon During Treatment

Lipolysis and oxidation are associated with the changes in fat during processing, wherein lipolysis is related to the catalysis of lipases. Table 5 displays that acid lipase had the highest activity among the three types of lipolytic enzymes during the treatment, followed by neutral lipase, while phospholipase showed the lowest activity. These results were in agreement with those of previous studies that reported the activity of lipases in smoke-cured bacon [8,11]. Another study indicated that neutral lipase had the highest activity in Jinhua ham [10]. During treatment, the activities of acid lipase and phospholipase continuously decreased with time throughout the treatment, consistent with a previous study [10]. However, another study [8] revealed that the activities of acid lipase and phospholipase in bacon increased and reached maximum values at day 10 of smoking treatment or remained stable during curing treatment. The activity of neutral lipase significantly increased from 0.324 to 0.727 during the curing stage, with the maximum value observed in C5d sample, and then significantly decreased (*p* < 0.05) from 0.727 to 0.167 until the end of the smoking treatment, showing results consistent with those of a previous study [11].

### 3.5. Changes in LOX Activity and Lipid Oxidation in Traditional Chinese Bacon During Treatment

As shown in Table 5, the LOX activity increased significantly from 16.01 to 30.34 in the curing stage due to the activating effect of salt, but it decreased to 6.28 at the end of the smoking stage, which might be associated with the effect of processing conditions or inactivation of hydroperoxides [8]. Previous studies have indicated that LOX shows strong activity throughout the smoking treatment of traditional Chinese bacon. Based on the results presented in Table 4, the levels of lipid oxidation products were measured. The POV and TBARS values varied based on the balance between the creation and breakdown of hydroperoxides, indicating the presence of primary and secondary products from lipid oxidation, respectively. There was a significant increase (*p* < 0.05) in both the POV and TBARS values at each stage of bacon processing, but no significant difference was found between samples S5d and S10d in terms of POV and MDA levels (*p* > 0.05). This lack of difference could be attributed to the antioxidant properties of certain phenolic compounds present in the smoking liquid [27,28].

### 3.6. Comparison of the Microstructures of Different Fat Portions of Bacon

**Figure 2:**
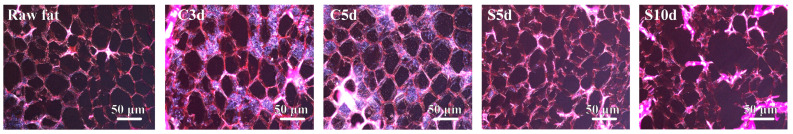
Polarized light microscopy of fat portion cell membrane morphology of bacon.

Adipocytes are surrounded by an extracellular matrix in fat tissue, which is primarily made up of two collagen-based structures: the basement membrane and interlobular septa [29,30]. The morphology of adipocyte membranes in traditional Chinese bacon was determined via polarized light microscopy (Figure 2). The diameter of a typical adipocyte ranges from 50 to 200 μm. In the curing stage, the integrity of the adipocyte membrane was better in the raw fat than in the fat portion of bacon; the adipocyte membrane was more permeable due to salt penetration, and tiny crystals appeared on the membrane. In the low-temperature smoking stage, a gradual disruption of the adipocyte membrane structure was clearly detected. The significant decrease in phospholipid contents also demonstrated that the adipocyte membrane was destroyed during processing. Overall, adipocyte membranes were broken down and decomposed, and the fat tissue was filled with a large number of lipid droplets accompanied by greatly reduced adipocyte membranes, resulting in the dissolution of fat crystals and fewer particles of the fat tissue after cooking. The transmittance of the fat tissue increased after cooking, thereby rendering it transparent.

## 4. Conclusions

Changes in the fat portion of bacon during LTS were investigated to determine the mechanism by which low-temperature smoking treatment contributes to the development of quality properties of the traditional Chinese fat portion of bacon. The results showed that the phospholipid content was significantly lower in the fat portion of bacon than in the raw fat and that there was no significant change in the SFA contents between the two groups. The DSC results also revealed that the changes in the melting point of the fat portion of bacon were consistent with those of SFAs. Changes in lipase content and oxidation of the fat fraction during processing were then investigated to further explain the changes in fat and fatty acid composition during treatment. Finally, polarized light microscopy revealed that the membranes of several adipocytes were disrupted and degraded during processing. In conclusion, the mechanism underlying the development of quality properties of fat portions of bacon is mainly related to the reduction of adipocyte membranes during processing and changes in fatty acid composition. The findings of our research offer a theoretical foundation for the advancement of innovative processing techniques for traditional Chinese bacon. In particular, we propose the substitution of traditional smoking methods with liquid smoke spray technology. This approach not only enhances the quality and safety of the product but also contributes to the promotion of environmentally sustainable processing methods.

## Figures and Tables

**Table 1:** Changes in color and texture of Chinese fat portion of bacon during LTS.

	Control	C3d	C5d	S5d	S10d
L*	78.82 ± 0.22 ^a^	77.09 ± 0.11 ^b^	74.22 ± 0.27 ^c^	64.90 ± 0.56 ^d^	52.45 ± 0.57 ^e^
a*	0.92 ± 0.30 ^a^	2.22 ± 0.22 ^b^	1.86 ± 0.11 ^b^	1.91 ± 0.30 ^b^	2.09 ± 0.09 ^b^
b*	6.02 ± 0.09 ^e^	7.07 ± 0.10 ^d^	7.70 ± 0.04 ^c^	8.52 ± 0.45 ^b^	15.08 ± 0.40 ^a^
Appearance Of fat	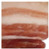	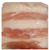	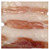	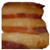	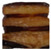
Appearance of traditional Chinese bacon	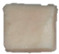	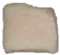	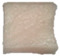	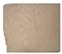	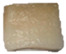
Hardness(g)	4558 ± 330.93 ^d^	5358 ± 233.15 ^d^	10,443 ± 624.14 ^c^	15,127 ± 533.20 ^b^	17,836 ± 815.34 ^a^
Springiness(mm)	0.91 ± 0.00 ^a^	0.94 ± 0.02 ^a^	0.81 ± 0.09 ^b^	0.78 ± 0.03 ^b^	0.68 ± 0.03 ^c^
Cohesiveness	0.63 ± 0.14 ^b^	0.79 ± 0.14 ^ab^	0.75 ± 0.05 ^ab^	0.61 ± 0.11 ^b^	0.87 ± 0.06 ^a^
Resilience	0.31 ± 0.09 ^c^	0.31 ± 0.06 ^c^	0.53 ± 0.04 ^b^	0.40 ± 0.09 ^c^	0.76 ± 0.07 ^a^

Note: Control—raw pork; C3d—the third day of the curing process; C5d—the fifth day of the curing process; S5d—the fifth day of the smoking process; S10d—the tenth day of the smoking process. Values are the M ± SD of three analyses. Different letters within the same row represent significant differences (*p* < 0.05).

**Table 2:** Changes in the lipid composition of Chinese fat portion of bacon during LTS.

	Control	C3d	C5d	S5d	S10d
Triacylglycerol (g/100 g total fat)	69.76 ± 1.13 ^d^	72.45 ± 1.67 ^c^	74.52 ± 1.28 ^c^	77.79 ± 1.41 ^b^	82.45 ± 1.43 ^a^
Phospholipid (g/100 g total fat)	26.2 ± 1.44 ^a^	21.74 ± 0.99 ^b^	18.63 ± 1.02 ^c^	13.64 ± 0.85 ^d^	6.59 ± 1.38 ^e^
Free fatty acids (g/100 g total fat)	4.04 ± 0.81 ^d^	5.81 ± 1.42 ^cd^	7.19 ± 1.34 b^c^	8.57 ± 0.83 ^b^	10.96 ± 0.86 ^a^

Note: Control—raw pork; C3d—the third day of the curing process; C5d—the fifth day of the curing process; S5d—the fifth day of the smoking process; S10d—the tenth day of the smoking process. Values are the M ± SD of three analyses. Different letters within the same row represent significant differences (*p* < 0.05).

**Table 3:** Changes in fatty acid composition (%) in fat during processing.

Fatty Acid	Control	C3d	C5d	S5d	S10d
C6:0	0.002 ± 0.000 a	0.001 ± 0.001 ^a^	0.001 ± 0.001 ^a^	0.002 ± 0.000 ^a^	0.001 ± 0.001 ^a^
C8:0	0.016 ± 0.000 ^a^	0.013 ± 0.000 ^b^	0.015 ± 0.001 ^a^	0.013 ± 0.001 ^b^	0.016 ± 0.001 ^a^
C10:0	0.128 ± 0.003 ^a^	0.107 ± 0.003 ^b^	0.112 ± 0.003 ^b^	0.107 ± 0.008 ^b^	0.133 ± 0.009 ^a^
C12:0	0.122 ± 0.001 ^a^	0.124 ± 0.003 ^a^	0.119 ± 0.013 ^a^	0.123 ± 0.001 ^a^	0.127 ± 0.004 ^a^
C14:0	1.808 ± 0.032 ^a^	1.618 ± 0.049 ^b^	1.609 ± 0.086 ^b^	1.687 ± 0.061 ^b^	1.907 ± 0.046 ^a^
C14:1	0.026 ± 0.000 ^b^	0.02 ± 0.002 ^c^	0.022 ± 0.001 ^bc^	0.023 ± 0.004 ^bc^	0.033 ± 0.003 ^a^
C15:0	0.035 ± 0.011 ^a^	0.053 ± 0.002 ^a^	0.044 ± 0.010 ^a^	0.049 ± 0.002 ^a^	0.040 ± 0.001 ^a^
C16:0	30.981 ± 0.225 ^a^	28.961 ± 0.22 ^c^	29.262 ± 0.335 ^c^	29.772 ± 0.265 ^b^	31.198 ± 0.309 ^a^
C16:1	4.014 ± 0.144 ^a^	3.166 ± 0.032 ^c^	3.347 ± 0.039 ^c^	3.627 ± 0.073 ^b^	4.201 ± 0.248 ^a^
C17:0	0.209 ± 0.022 ^bc^	0.276 ± 0.010 ^a^	0.235 ± 0.051 ^abc^	0.253 ± 0.014 ^ab^	0.201 ± 0.002 ^c^
C17:1	0.31 ± 0.042 ^a^	0.291 ± 0.005 ^ab^	0.263 ± 0.048 ^ab^	0.276 ± 0.008 ^ab^	0.247 ± 0.002 ^b^
C18:0	13.045 ± 0.519 ^a^	13.717 ± 0.209 ^a^	13.014 ± 0.174 ^a^	13.443 ± 0.534 ^a^	12.959 ± 0.598 ^a^
C18:1	26.845 ± 0.395 ^b^	26.084 ± 0.168 ^c^	26.859 ± 0.180 ^b^	26.612 ± 0.302 ^b^	27.989 ± 0.103 ^a^
C18:2 trans	0.158 ± 0.007 ^b^	0.125 ± 0.007 ^c^	0.132 ± 0.001 ^c^	0.131 ± 0.018 ^c^	0.186 ± 0.011 ^a^
C18:2	18.363 ± 0.313 ^c^	21.162 ± 0.349 ^a^	21.029 ± 0.041 ^a^	19.675 ± 0.185 ^b^	16.767 ± 0.068 ^d^
C18:3 n6	0.037 ± 0.013 ^b^	0.058 ± 0.002 ^a^	0.050 ± 0.010 ^ab^	0.050 ± 0.001 ^ab^	0.046 ± 0.004 ^ab^
C18:3	0.878 ± 0.018 ^ab^	1.039 ± 0.026 ^a^	0.914 ± 0.204 ^ab^	0.959 ± 0.022 ^ab^	0.817 ± 0.003 ^b^
C20:0	0.275 ± 0.024 ^ab^	0.241 ± 0.010 ^c^	0.242 ± 0.016 ^c^	0.255 ± 0.007 ^bc^	0.286 ± 0.010 ^a^
C20:1 n9	1.014 ± 0.015 ^ab^	0.967 ± 0.029 ^b^	0.961 ± 0.027 ^b^	1.058 ± 0.029 ^a^	1.090 ± 0.083 ^a^
C20:2	0.879 ± 0.009 ^ab^	0.979 ± 0.027 ^a^	0.847 ± 0.146 ^ab^	0.972 ± 0.019 ^ab^	0.843 ± 0.030 ^b^
C21:0	0.027 ± 0.005 ^b^	0.036 ± 0.003 ^a^	0.034 ± 0.004 ^ab^	0.032 ± 0.003 ^ab^	0.035 ± 0.006 ^ab^
C20:3 n6	0.120 ± 0.031 ^b^	0.153 ± 0.004 ^a^	0.143 ± 0.019 ^ab^	0.143 ± 0.001 ^ab^	0.141 ± 0.007 ^ab^
C20:4 n6	0.408 ± 0.048 ^a^	0.429 ± 0.009 ^a^	0.407 ± 0.034 ^a^	0.390 ± 0.008 ^a^	0.418 ± 0.044 ^a^
C20:3 n3	0.115 ± 0.028 ^b^	0.156 ± 0.008 ^a^	0.138 ± 0.029 ^ab^	0.158 ± 0.005 ^a^	0.132 ± 0.010 ^ab^
C22:0	0.002 ± 0.000 ^b^	0.002 ± 0.001 ^b^	0.002 ± 0.000 ^b^	0.003 ± 0.000 ^a^	0.003 ± 0.000 ^a^
C22:1	0.014 ± 0.000 ^bc^	0.015 ± 0.000 ^b^	0.013 ± 0.001 ^c^	0.014 ± 0.002 ^bc^	0.019 ± 0.000 ^a^
C22:2	0.004 ± 0.002 ^b^	0.007 ± 0.001 ^a^	0.006 ± 0.001 ^ab^	0.005 ± 0.000 ^b^	0.006 ± 0.001 ^ab^
C23:0	0.014 ± 0.000 ^a^	0.012 ± 0.001 ^b^	0.010 ± 0.002 ^bc^	0.011 ± 0.000 ^b^	0.009 ± 0.001 ^c^
C24:0	0.113 ± 0.016 ^b^	0.142 ± 0.007 ^a^	0.123 ± 0.020 ^ab^	0.119 ± 0.001 ^b^	0.116 ± 0.006 ^b^
C22:6 n3	0.038 ± 0.000 ^ab^	0.047 ± 0.001 ^a^	0.046 ± 0.010 ^a^	0.039 ± 0.006 ^ab^	0.032 ± 0.001 ^b^
SFA	46.778 ± 0.739 ^a^	45.304 ± 0.097 ^bc^	44.823 ± 0.346 ^c^	45.87 ± 0.207 ^b^	47.032 ± 0.226 ^a^
MUFA	32.222 ± 0.567 ^b^	30.543 ± 0.171 ^d^	31.465 ± 0.065 ^c^	31.609 ± 0.417 ^bc^	33.580 ± 0.269 ^a^
PUFA	20.881 ± 0.202 ^c^	23.990 ± 0.276 ^a^	23.569 ± 0.381 ^a^	22.359 ± 0.205 ^b^	19.250 ± 0.034 ^d^
PUFA/SFA	0.446 ± 0.012 ^c^	0.530 ± 0.008 ^a^	0.526 ± 0.013 ^a^	0.487 ± 0.003 ^b^	0.409 ± 0.002 ^d^

Note: Control—raw pork; C3d—the third day of the curing process; C5d—the fifth day of the curing process; S5d—the fifth day of the smoking process; S10d—the tenth day of the smoking process. SFA—monounsaturated fatty acid; UFA—unsaturated fatty acid; MUFA—monounsaturated fatty acid; PUFA—polyunsaturated fatty acid; PUFA/SFA—the ratio of polyunsaturated fatty acids to monounsaturated fatty acids. Values are the M ± SD of three analyses. Different letters within the same row represent significant differences (*p* < 0.05).

**Table 4:** Crystallization temperature, melting temperature and enthalpy of fat portion of bacon during LTS.

		Control	C3d	C5d	S5d	S10d
Melt	T1M (°C)	−26.63 ± 0.21 ^d^	−25.70 ± 0.33 ^c^	−24.77 ± 0.15 ^b^	−24.35 ± 0.23 ^ab^	−24.01 ± 0.40 ^a^
	T2M (°C)	16.45 ± 0.26 ^a^	15.35 ± 0.21 ^bc^	14.58 ± 0.43 ^c^	15.70 ± 0.27 ^b^	15.20 ± 0.64 ^bc^
	T3M (°C)	32.09 ± 0.22 ^a^	31.53 ± 0.45 ^a^	-	-	-
	ΔM, Total (J/g)	53.13 ± 0.31 ^d^	58.11 ± 0.60 ^ab^	55.90 ± 0.86 ^c^	57.72 ± 0.31 ^b^	58.70 ± 0.25 ^a^
Crystal	T1C (°C)	10.49 ± 0.73 ^a^	9.59 ± 0.25 ^b^	8.54 ± 0.44 ^c^	-	-
	T2C (°C)	6.37 ± 0.34 ^c^	6.45 ± 0.33 ^c^	4.23 ± 0.51 ^d^	11.30 ± 0.31 ^b^	13.93 ± 0.38 ^a^
	T3C (°C)	−11.37 ± 0.85 ^b^	−9.55 ± 0.72 ^a^	−12.52 ± 0.37 ^c^	−11.26 ± 0.47 ^b^	−10.01 ± 0.21 ^a^
	ΔC, Total (J/g)	61.38 ± 0.38 ^c^	64.55 ± 0.47 ^a^	62.63 ± 1.01 ^b^	64.61 ± 0.32 ^a^	62.78 ± 0.73 ^b^

Note: Control—raw pork; C3d—the third day of the curing process; C5d—the fifth day of the curing process; S5d—the fifth day of the smoking process; S10d—the tenth day of the smoking process. T1C, T2C, T3C are the starting temperatures of the 1st, 2nd, and 3rd occurring crystallization peaks, respectively.T1M, T2M, T3M are the starting temperatures of the 1st, 2nd, and 3rd occurring melting peaks, respectively. ΔC—Total represents the exothermic enthalpy of crystallization. ΔM—Total represents the enthalpy of heat absorption by melting. - indicates not detected. Values are the M ± SD of three analyses. Different letters within the same row represent significant differences (*p* < 0.05).

**Table 5:** Changes in lipase activity and oxidation of fat portion of bacon during LTS.

	Control	C3d	C5d	S5d	S10d
Neutral lipase	0.32 ± 0.01 ^c^	0.55 ± 0.03 ^b^	0.73 ± 0.03 ^a^	0.23 ± 0.03 ^c^	0.17 ± 0.02 ^d^
Phospholipase	0.78 ± 0.05 ^a^	0.59 ± 0.02 ^b^	0.27 ± 0.02 ^c^	0.15 ± 0.02 ^d^	0.13 ± 0.02 ^d^
Acid lipase	4.84 ± 0.04 ^a^	3.13 ± 0.09 ^b^	2.96 ± 0.05 ^c^	1.65 ± 0.01 ^d^	1.54 ± 0.03 ^e^
Lipoxygenase	16.01 ± 0.41 ^e^	23.74 ± 1.14 ^b^	30.34 ± 0.24 ^a^	10.06 ± 0.40 ^c^	6.28 ± 0.68 ^d^
POV (milliequivalent peroxide/kg lipid)	0.01 ± 0.00 ^d^	0.08 ± 0.00 ^c^	0.09 ± 0.00 ^b^	0.13 ± 0.01 ^a^	0.13 ± 0.00 ^a^
TBARS (mg MDA equivalents/Kg fat)	0.07 ± 0.01 ^d^	0.10 ± 0.02 ^c^	0.11 ± 0.01 ^b^	0.14 ± 0.00 ^a^	0.15 ± 0.01 ^a^

Note: Control—raw pork; C3d—the third day of the curing process; C5d—the fifth day of the curing process; S5d—the fifth day of the smoking process; S10d—the tenth day of the smoking process. Values are the M ± SD of three analyses. Different letters within the same row represent significant differences (*p* < 0.05).

## Data Availability

The original contributions presented in the study are included in the article, further inquiries can be directed to the corresponding authors.
